# Dlx3b/4b is required for early-born but not later-forming sensory hair cells during zebrafish inner ear development

**DOI:** 10.1242/bio.026211

**Published:** 2017-07-27

**Authors:** Simone Schwarzer, Sandra Spieß, Michael Brand, Stefan Hans

**Affiliations:** Technische Universität Dresden, Biotechnology Center and DFG-Center for Regenerative Therapies Dresden Cluster of Excellence, Tatzberg 47-49, 01307 Dresden, Germany

**Keywords:** Inner ear, Neurogenesis, Sensorigenesis, Tether cells, Dlx3b/4b, Foxi1, CRISPR/Cas9, Zebrafish

## Abstract

Morpholino-mediated knockdown has shown that the homeodomain transcription factors Dlx3b and Dlx4b are essential for proper induction of the otic-epibranchial progenitor domain (OEPD), as well as subsequent formation of sensory hair cells in the developing zebrafish inner ear. However, increasing use of reverse genetic approaches has revealed poor correlation between morpholino-induced and mutant phenotypes. Using CRISPR/Cas9-mediated mutagenesis, we generated a defined deletion eliminating the entire open reading frames of *dlx3b* and *dlx4b* (*dlx3b/4b*) and investigated a potential phenotypic difference between mutants and morpholino-mediated knockdown. Consistent with previous findings obtained by morpholino-mediated knockdown of Dlx3b and Dlx4b, *dlx3b/4b* mutants display compromised otic induction, the development of smaller otic vesicles and an elimination of all indications of otic specification when combined with loss of *foxi1*, a second known OEPD competence factor in zebrafish. Furthermore, sensorigenesis is also affected in *dlx3b/4b* mutants. However, we find that only early-born sensory hair cells (tether cells), that seed and anchor the formation of otoliths, are affected. Later-forming sensory hair cells are present, indicating that two genetically distinct pathways control the development of early-born and later-forming sensory hair cells. Finally, impairment of early-born sensory hair cell formation in *dlx3b/4b* mutant embryos reverses the common temporal sequence of neuronal and sensory hair cell specification in zebrafish, resembling the order of cell specification in amniotes; *Neurog1* expression before *Atoh1* expression. We conclude that the Dlx3b/4b-dependent pathway has been either acquired newly in the fish lineage or lost in other vertebrate species during evolution, and that the events during early inner ear development are remarkably similar in fish and amniotes in the absence of this pathway.

## Abstract (precis)

**Summary:** The transcription factors Dlx3b and Dlx4b control the formation of early-born sensory hair cells or tether cells in the developing zebrafish inner ear.

## INTRODUCTION

The vertebrate inner ear is a sensory organ mediating hearing and balance. It derives from the otic placode, a transient ectodermal thickening adjacent to the developing hindbrain, and contains a complex arrangement of mechanosensory hair cells, nonsensory supporting cells and sensory neurons (Barald and Kelley, 2004; Raft and Groves, 2015; Whitfield, 2015). Inner ear formation is a multistep process initiated by the establishment of the preplacodal region, a zone of ectoderm running around the anterior border of the neural plate containing precursors for all sensory placodes (Streit, 2007). The preplacodal region is further specified into a common otic-epibranchial progenitor domain (OEPD) that in zebrafish also contains the progenitors of the anterior lateral line ganglion (Chen and Streit, 2013; McCarroll et al., 2012; Hans et al., 2013). Signaling molecules of the fibroblast growth factor (Fgf) and Wnt/wingless families are critical for OEPD induction and differentiation in all vertebrates examined to date (Phillips et al., 2001; Léger and Brand, 2002; Maroon et al., 2002; Solomon and Fritz, 2002; Alvarez et al., 2003; Solomon et al., 2003; Wright and Mansour, 2003; Ladher et al., 2005; Freter et al., 2008; McCarroll et al., 2012; Ladher, 2017). In amniotes, competence to respond to Fgf signals is conferred by Foxi3, a forkhead transcription factor, whereas in zebrafish, competence is provided by the functional homolog Foxi1 and additionally by the distal-less homeodomain transcription factors Dlx3b and Dlx4b (Dlx3b/4b) (Hans et al., 2004; Solomon et al., 2004; Yamanishi et al., 2012; Khatri et al., 2014; Birol et al., 2016). The otic placode develops into the otocyst or otic vesicle with mechanosensory hair cells generated in the sensory epithelia and neuronal precursors delaminating as neuroblasts from the ventral face of the otic vesicle (Haddon and Lewis, 1996; Rubel and Fritzsch, 2002). Both lineages require the activity of the proneural proteins Atonal homologue1 (Atoh1) and Neurogenin1 (Neurog1) (Ma et al., 1998; Bermingham et al., 1999; Andermann et al., 2002; Fritzsch et al., 2010). Recent results indicate that Foxi1 and Dlx3b/4b act upstream of Neurog1 and Atoh1, respectively, implying transcriptional inputs for neural and sensory cell formation to OEPD stages in zebrafish (Hans et al., 2013). Notably, the temporal specification of neuroblasts and hair cells is not constrained across phyla, and is reversed in zebrafish compared with amniotes. In mouse, expression of *Neurog1* in the developing inner ear occurs at embryonic day (E) 8.75 preceding *Atoh1* expression at E10.5 (Fritzsch et al., 2010). In zebrafish, *atoh1b* is initiated at 10.5 h postfertilization (hpf) followed by *atoh1a* at 14 hpf (Millimaki et al., 2007), preceding otic *neurog1* expression at 15 hpf (Radosevic et al., 2014). The reasoning behind this reversal is currently unknown. In zebrafish, the first hair cells can be observed already at 20 hpf, whereas neuronal precursors arise between 22 to 42 hpf (Haddon and Lewis, 1996; Riley et al., 1997). Early-born hair cells form in an *atoh1b*-dependent manner and give rise to tether cells that seed and anchor the formation of otoliths, large solidified bio-crystals that mediate vestibular function (Millimaki et al., 2007). Recent results predict the existence of a hair cell-specific otolith precursor-binding factor, but the identity of such a factor remains elusive (Stooke-Vaughan et al., 2012). In contrast, amniotes do not form otoliths, but structurally similar bio-crystals called otoconia, located above hair cells of the saccule and utricle, enable detection of gravity and linear acceleration (Lundberg et al., 2015).

In zebrafish, knockdown is easily achieved via antisense morpholino oligonucleotides, which possess a modified backbone providing protection from degradation and bind close to the translational start site or at splice sites, thus blocking translation or splicing, respectively (Bill et al., 2009). However, an increasing number of gene knockout studies using programmable nucleases have revealed discrepancies with published morpholino-induced phenotypes (Kok et al., 2015; Novodvorsky et al., 2015; Boer et al., 2016). In particular, in a large collection of knockout lines, >70% of morpholino-induced phenotypes failed to be observed in the corresponding mutants (Kok et al., 2015). This suggests that morpholino-mediated off-target effects are much more prevalent than previously stated, and highlight the need to re-evaluate the use of antisense-based technology for characterization of gene function in the zebrafish. Here, we revisited the function of Dlx3b/4b during zebrafish OEPD development using a newly generated null mutant carrying a defined deletion of *dlx3b* and *dlx4b* (*dlx3b/4b*). We show that *dlx3b/4b* mutants confirm previous findings generated by morpholino-mediated knockdown with respect to compromised otic induction and the development of smaller otic vesicles. Furthermore, combined loss of *dlx3b/4b* and *foxi1* eliminates all indications of otic specification at all stages examined. Additionally, we confirm that Dlx3b/4b is required for proper formation of sensory hair cells. However, we find that *dlx3b/4b* mutants only impair the formation of early-born hair cells (also known as tether cells) required for seeding and anchoring the formation of otoliths, whereas later-forming sensory hair cells are present. This indicates that two genetically distinct pathways result in the formation of early-born and later-forming sensory hair cells, respectively. Furthermore, loss of early-born hair cells in *dlx3b/4b* mutant embryos reverses the temporal order of neuronal and hair cell specification, resembling the order of cell specification in amniotes, which express *Neurog1* before *Atoh1*. In summary, we conclude that the Dlx3b/4b-dependent pathway has either been acquired in the fish lineage or lost in other vertebrate lineages and that inner ear development is highly similar in amniotes and fish in the absence of this pathway.

## RESULTS

### Generation of a defined chromosomal deletion at the *dlx3b* and *dlx4b* locus

**Fig. 1.**
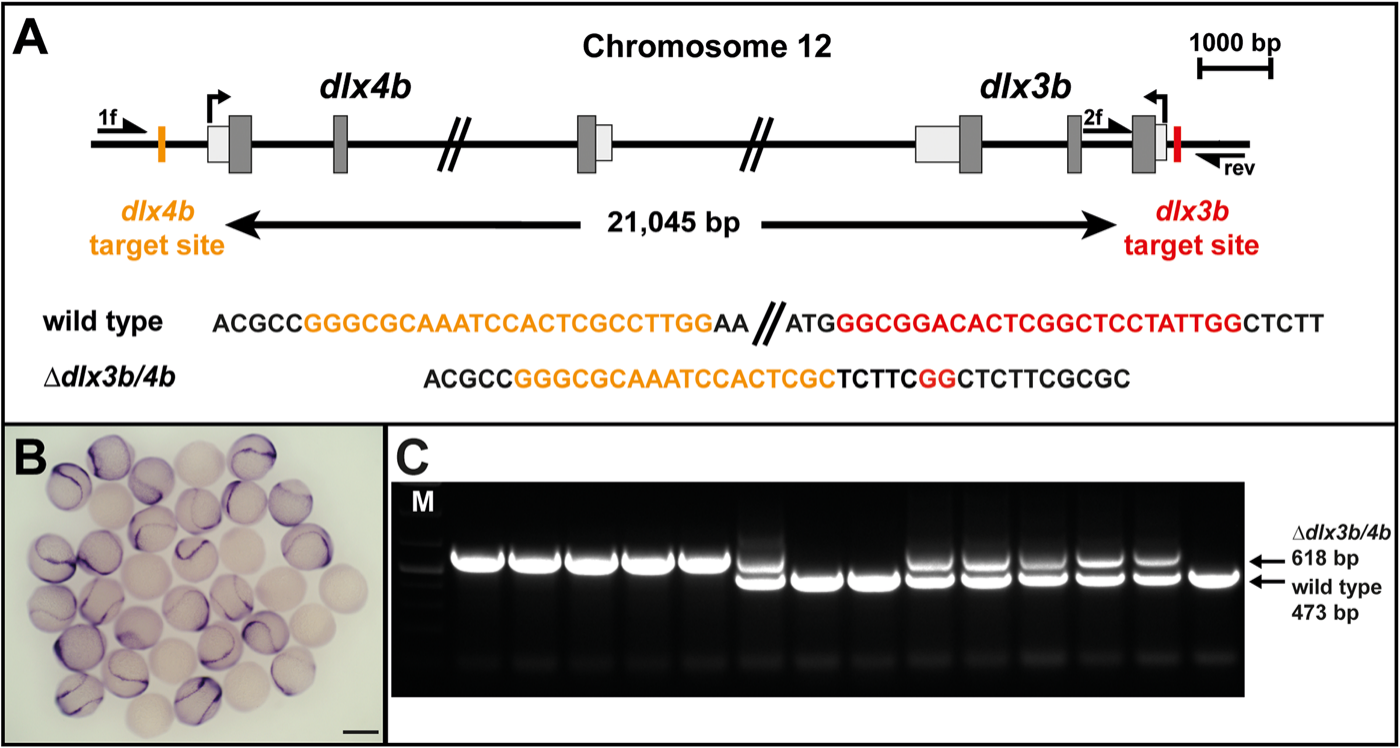
**Generation of a *dlx3b* and *dlx4b* (*dlx3b/4b*) null allele.** (A) Scheme of the *dlx3b/4b* bigene cluster at chromosome 12. Transcriptional start sites are indicated by arrows. Exon sequences with translated and untranslated regions are represented in dark and light grey, respectively. Positions of the CRISPR/Cas9 target sequences, separated by 21,045 bp, and their sequences in the wild-type and *dlx3b/4b* deletion allele are indicated in orange and red. Primers used for genotyping (1f, 2f and rev) are shown as half arrows. (B) *In situ* hybridization of *dlx3b* at early OEPD stages (three somites). Expression of *dlx3b* is absent in 25% of embryos obtained from a *dlx3b/4b* heterozygote incross. Scale bar: 500 µm. (C) Multiplex PCR using primers 1f, 2f and rev reveals the presence of the *dlx3b/4b* deletion (618 bp) and wild-type allele (473 bp). M indicates a marker for molecular size standard.

We envisioned that the best strategy to generate an unambiguous null allele would be to introduce a defined deletion eliminating the entire open reading frame in the locus of interest. This approach is particularly useful for avoiding alternative translational start sites and allows easy identification of carrier fish. To achieve a simultaneous deletion of *dlx3b* and *dlx4b*, which are organized in a tail-to-tail bigene cluster (*dlx3b/4b*), two CRISPR/Cas9 target sites separated by 21,045 bp up- and downstream from the cluster were chosen (Fig. 1A). Injection of gRNA that targeted only a single site along with mRNA encoding a dual NLS-tagged zebrafish codon-optimized Cas9 protein into single-cell zebrafish embryos resulted in site-specific insertion/deletion (indel) mutations in the corresponding target sites as evaluated by polymerase chain reaction (PCR) and sequencing (data not shown). After co-injection of both gRNAs, deletion events in the genomic region between the two target sites were detected by PCR (primer 1f and rev), which were confirmed by sequencing (data not shown). Injected siblings were grown to adulthood and tested for germ-line transmission of the deletion to F_1_ fish using the previously established PCR strategy. Among the 80 animals examined, one founder was identified carrying the deletion of the entire *dlx3b/4b* locus was established (Fig. 1A). *In situ* hybridization of embryos at early segmentation stages obtained from incrosses of heterozygous carriers showed a complete loss of *dlx3b* mRNA in 25% of the clutch, corroborating the absence of the *dlx3b* gene (Fig. 1B). In order to enable accurate genotyping of progeny from incrosses of heterozygous carriers, we established a multiplex PCR strategy by adding a second forward primer (f2), which anneals to genomic sequences absent in the *dlx3b/4b* deletion allele (Fig. 1A). Genotyping of five embryos selected at 24 hpf that displayed an obvious phenotype (see below) proved to harbor only the *dlx3b/4b* deletion allele indicated by the presence of a 618-bp amplicon (Fig. 1C). In contrast, nine randomly selected embryos with wild-type morphology contained the wild-type allele shown by the presence of a 473-bp fragment either in homozygosity or in combination with the *dlx3b/4b* deletion allele (Fig. 1C). Taken together, CRISPR/Cas9-mediated mutagenesis resulted in the predicted generation of a *dlx3b/4b* null allele.

### Loss of Dlx3b/4b results in compromised OEPD induction and subsequent otic vesicle hypoplasia

**Fig. 2.**
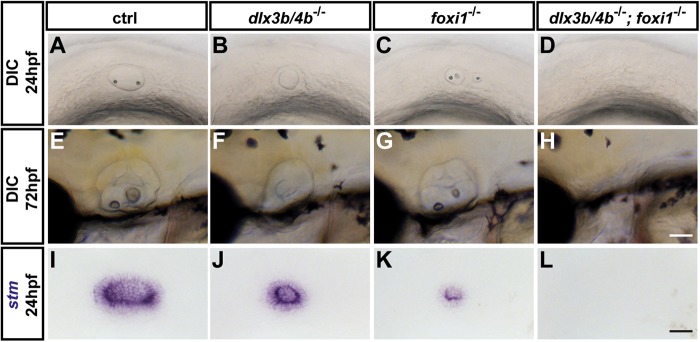
**Loss of Dlx3b/4b results in otic vesicle hypoplasia.** (A-H) Live images of wild-type control embryos (A,E), *dlx3b/4b* mutants (B,F), *foxi1* mutants (C,G) and *dlx3b/4b*; *foxi1* double mutants (D,H) at 24 hpf (A-D) and 72 hpf (E-H). (I-L) Expression of *starmaker* (*stm*), a marker of the otic epithelium in wild-type control siblings (I), *dlx3b/4b* mutants (J), *foxi1* mutants (K) and *dlx3b/4b*; *foxi1* double mutants (L) at 24 hpf. Lateral views are seen with anterior to the left. Scale bars: 50 µm in H; 40 µm in L.

**Fig. 3.**
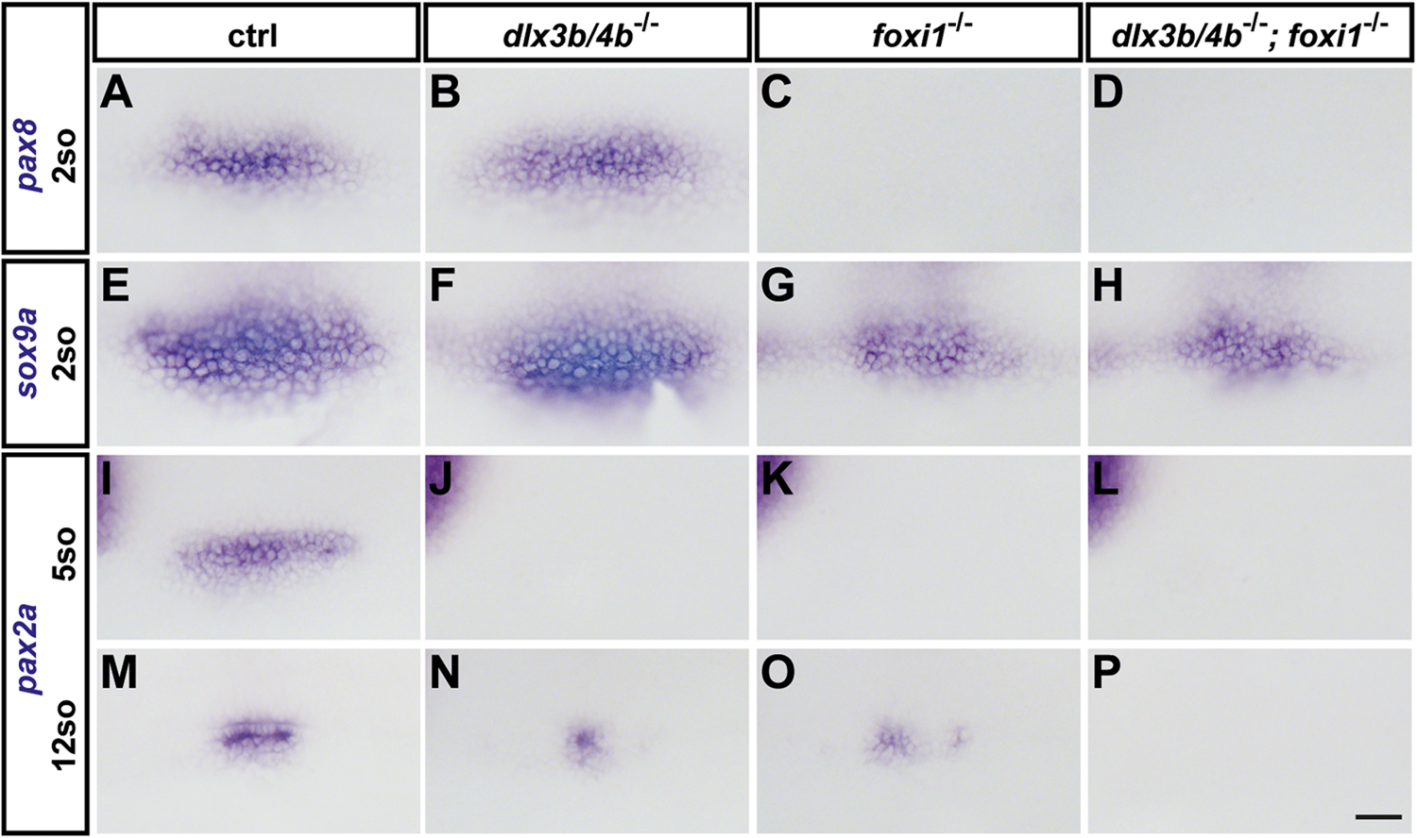
**Loss of Dlx3b/4b results in compromised OEPD induction.** *In situ* hybridization of *pax8* (A-D), *sox9a* (E-H) and *pax2a* (I-P) at early OEPD (two and five somites) and placodal (12 somites) stages in wild-type control embryos (A,E,I,M), *dlx3b/4b* mutants (B,F,J,N), *foxi1* mutants (C,G,K,O) and *dlx3b/4b*; *foxi1* double mutants (D,H,L,P). (A-L) Dorsal views with anterior to the left. (M-P) Dorsolateral views with anterior to the left. Scale bar: 40 µm.

Previously, morpholino-mediated knockdown of Dlx3b/4b has been reported to result in improper OEPD induction and subsequent compromised otic placode/vesicle formation (Solomon and Fritz, 2002). Indeed, incrosses of identified heterozygous *dlx3b/4b* deletion carriers confirmed previous findings and resulted in embryos with smaller otic vesicles, which were also demonstrated by starmaker (stm) expression, a marker of the entire otic epithelium (Fig. 2A,B,I,J). Reduction of otic tissue was partially rescued during subsequent development, but otolith formation remained defective in *dlx3b/4b* mutants compared with wild-type siblings (Fig. 2E,F). Furthermore, whereas *foxi1* mutants display reduced otic induction with highly variable otic vesicle morphologies (Nissen et al., 2003; Solomon et al., 2003), analysis of *dlx3b/4b;foxi1* double mutants showed that combined loss of both factors results in a complete absence of otic tissue (Fig. 2C,D,G,H,K,L), corroborating previous findings using morpholino-mediated knockdown of Dlx3b/4b (Hans et al., 2004; Solomon et al., 2004). Also consistent with previous findings, expression of *pax8*, the earliest OEPD marker, which is completely dependent on Foxi1, was unchanged in *dlx3b/4b* mutants in comparison with wild-type siblings (Fig. 3A-C). Similarly, *sox9a*, another early OEPD marker, which is partially dependent on Foxi1 activity, was also unchanged in *dlx3b/4b* mutants (Fig. 3E-G), contradictory to results reporting reduced expression of *sox9a* in the OEPD after morpholino-mediated knockdown of Dlx3b/4b (Liu et al., 2003). In contrast, *pax2a* was absent at early OEPD stages in both *dlx3b/4b* and *foxi1* mutants in comparison with control siblings, but recovered at late OEPD stages, and *pax2a* cDNA probes labeled size-reduced otic placodes in both mutant backgrounds (Fig. 3I-K,M-O and data not shown). Expression of *pax8*, *sox9a* and early OEPD *pax2a* in *dlx3b/4b;foxi1* double-mutant embryos mimicked findings in single mutants, but placodal *pax2a* expression failed to form, foreshadowing the complete absence of otic specification (Fig. 3D,H,L,P). Combined and with the exception of an effect of *sox9a* on early OEPD expression, these results show a strong correlation between morpholino-induced and mutant phenotypes of *dlx3b/4b,* and indicate that the previously used morpholinos represent a reliable tool to knock down *dlx3b/4b* gene function.

### Loss of early-born, but not later-forming, sensory hair cells in *dlx3b/4b* mutants

**Fig. 4.**
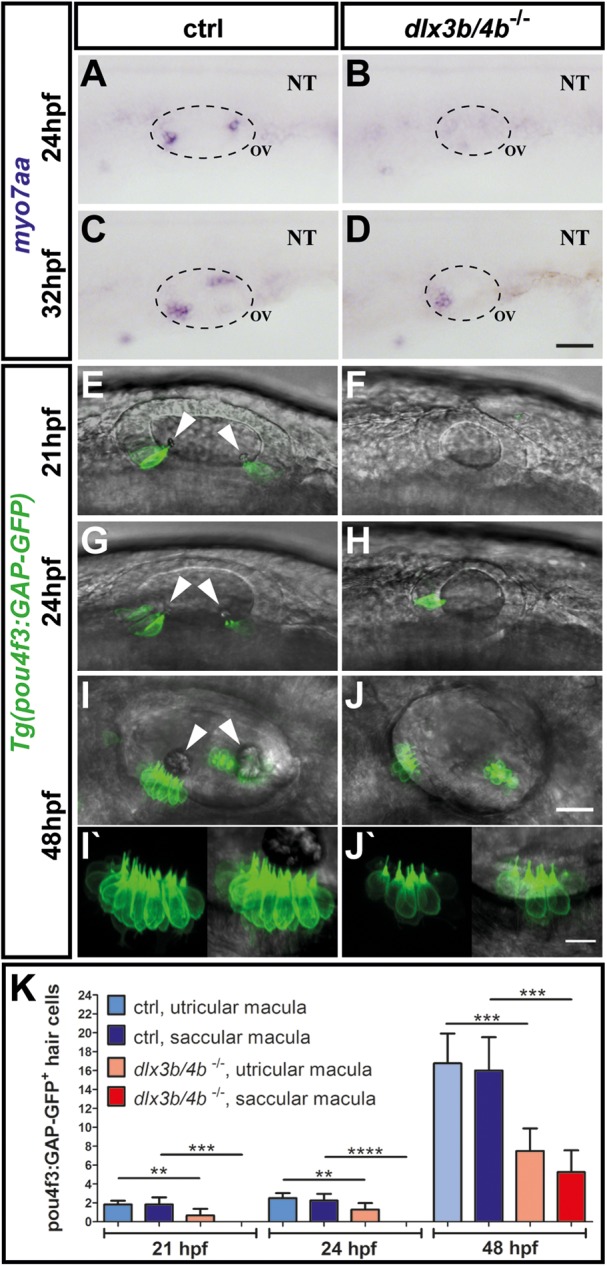
**Delayed formation of sensory hair cells in *dlx3b/4b*-deficient embryos.** (A-D) In comparison with control embryos at 24 hpf and 32 hpf, *myo7aa* expression is initially absent and subsequently restricted to the domain of the future utricular macula in *dlx3b/4b* mutants. (E-J) Expression of *Tg(pou4f3:GAP-GFP)* in control siblings and *dlx3b/4b* mutants at 21 hpf, 24 hpf and 48 hpf confirms delayed formation of sensory hair cells in *dlx3b/4b*-deficient embryos. Arrowheads indicate positions of the nascent otoliths in wild-type embryos. Note the initial onset of GFP in the prospective domain of the utricular macula and the subsequent expression in the future domain of the saccular macula in *dlx3b/4b* mutants. (I′,J′) Kinocilia formation appears normally in control siblings and *dlx3b/4b* mutants. Lateral views are shown with anterior to the left. NT, neural tube; OV, otic vesicle. Scale bars: 40 µm in D; 25 µm in J; 10 µm in J′. (K) Time course showing the mean number of pou4f3:GAP-GFP-positive hair cells in the domain of the future utricular and saccular domain of control (ctrl) and *dlx3b/4b* mutant embryos. *dlx3b/4b*-deficient embryos exhibit a significantly reduced number of sensory hair cells at 21 hpf and 24 hpf, and display significantly fewer hair cells at 48 hpf (**P<0,01; ***P<0,001; ****P<0,0001). Data are mean±s.e.m (n≥6 for each time point).

**Fig. 5.**
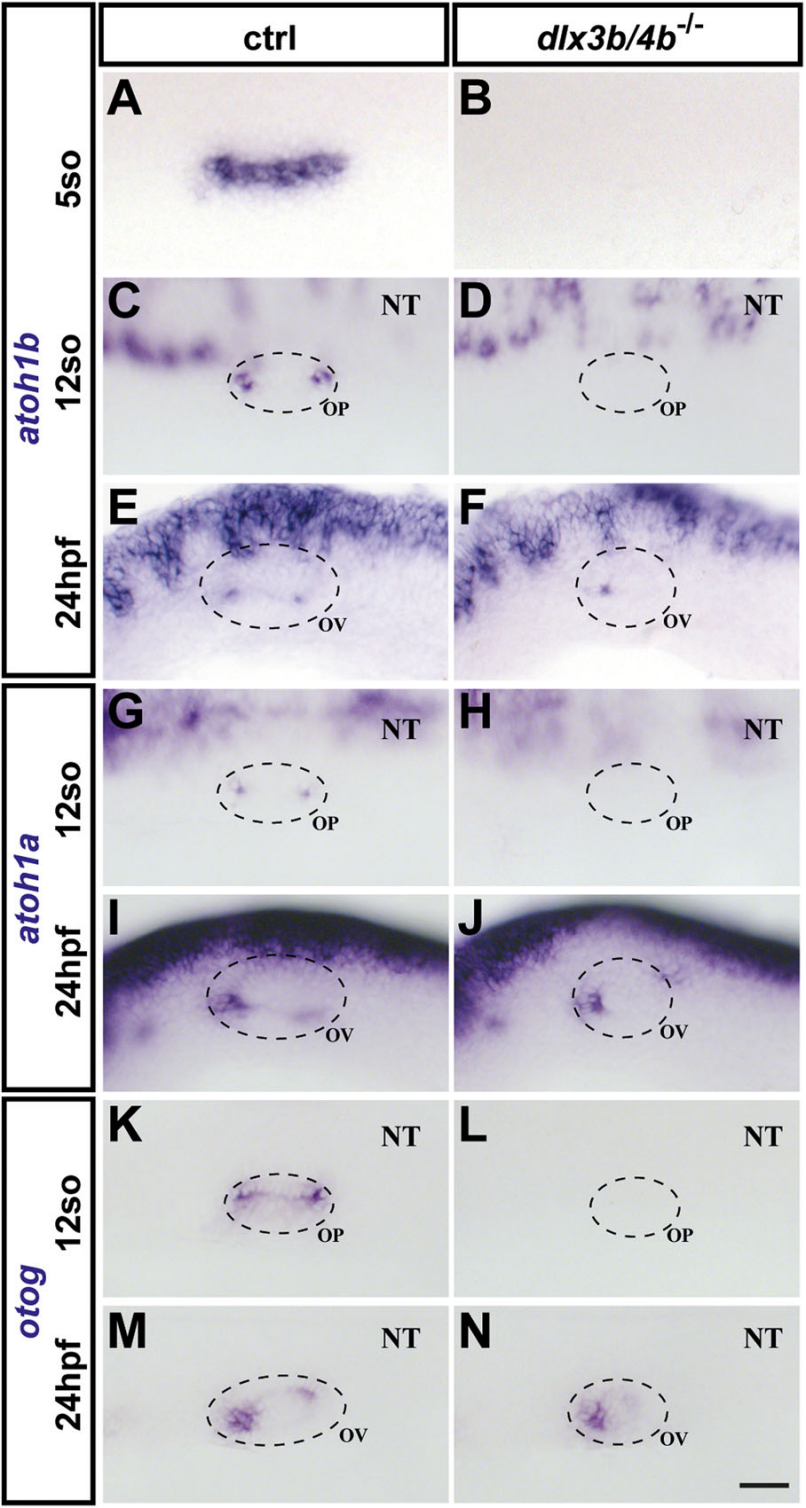
**Analysis of upstream regulators of sensory hair cell and otolith development.** (A-D) At OEPD (five somites) and placodal stages (12 somites), otic expression of *atoh1b* is absent in *dlx3b/4b* mutant embryos in comparison with wild-type siblings. (E,F) In contrast, *atoh1b* can be detected in *dlx3b/4b* mutants at 24 hpf although limited to the anterior portion of the otic vesicle. (G-J) Similarly, *atoh1a* is initially not expressed in the developing otic placode of *dlx3b/4b*-deficient embryos but present in the future domain of the utricular macula at 24 hpf. (K-N) Compared with wild-type control, *otog* expression is undetectable in *dlx3b/4b* mutants at placodal stages and restricted to the prospective domain of the utricular macula at 24 hpf. (A-D,G,H) Dorsolateral views with anterior to the left. (E,F,I,J) Lateral views with anterior to the left. OP, otic placode. Scale bar: 40 µm.

Previous studies have shown that Dlx3b/4b is required for proper expression of *atoh1a* and *atoh1b* and subsequent formation of mechanosensory hair cells (Millimaki et al., 2007; Hans et al., 2013). In addition, although morpholino-mediated knockdown of Dlx3b/4b results in a fully penetrant phenotype with complete failure in otolith development, formation of hair cells at later stages has been reported (Solomon and Fritz, 2002; Liu et al., 2003). This discrepancy could be explained either by insufficient supply of *dlx3b/4b* morpholino during injection, degradation of *dlx3b/4b* morpholino during subsequent development, or by the formation of sensory hair cells in a Dlx3b/4b-independent manner. This question can be addressed only by the production of a mutant allele. Hence, we analyzed sensory lineage development using *myosin VIIAa* (*myo7aa*), a marker of sensory hair cells (Ernest et al., 2000). Consistent with previous reports using morpholino knockdown (Millimaki et al., 2007; Hans et al., 2013), *dlx3b/4b* mutant embryos displayed a complete loss of *myo7aa* in comparison with control embryos, which expressed *myo7aa* at 24 hpf in discrete anterior and posterior domains of the otic vesicle corresponding to the prospective utricular and saccular maculae (Fig. 4A,B). At 32 hpf, the number of mature hair cells in the two sensory patches had increased in control embryos. Expression of *myo7aa* could now also be detected in the prospective utricular domain of the otic vesicle in *dlx3b/4b* mutants (Fig. 4C,D). To corroborate this finding, we analyzed expression of *Tg(pou4f3:GAP-GFP,)* which is a GFP marker for all differentiated hair cells of the developing otic vesicle (Xiao et al., 2005). In wild-type siblings at 21 hpf, GFP fluorescence was present in early-born hair cells or tether cells (which form in pairs at the anterior and posterior ends of the otocyst), whereas GFP could not be detected in otic vesicles of *dlx3b/4b* mutants (Fig. 4E,F). Slightly later, at 24 hpf, GFP expression was initiated in otic vesicles of *dlx3b/4b* mutants, but was initially restricted to the prospective utricular domain (Fig. 4G,H). Subsequently, additional hair cells formed also in the domain marking the future saccular maculae of *dlx3b/4b* mutants (Fig. 4I,J). Higher magnifications showed that hair cell kinocilia formation appeared to occur normally (Fig. 4I′,J′). However, quantification revealed that the total number of GFP-positive hair cells remained significantly lower in *dlx3b/4b* mutant embryos in comparison with control siblings (Fig. 4K). Previous studies have shown that early-born hair cells are generated in an *atoh1b*-dependent manner (Millimaki et al., 2007; Stooke-Vaughan et al., 2012). Consistently, *atoh1b* expression in the OEPD or otic placode was never observed in *dlx3b/4b* mutants compared with control embryos (Fig. 5A-D). However, at 22 hpf, *atoh1b* expression was initiated in *dlx3b/4b* mutants and present during subsequent vesicle stages, although confined to the domain of the future utricular macula (Fig. 5E,F). Similarly, *atoh1a* expression was absent in *dlx3b/4b* mutants, but present in wild-type embryos at placodal stages (Fig. 5G,H). Expression of *atoh1a* was activated prior to *atoh1b* at 20 hpf and continued to be expressed in otic vesicles at 24 hpf in *dlx3b/4b* mutants. Compared with control siblings at 24 hpf with *atoh1a* expression in the prospective utricular and saccular maculae, *atoh1a* expression was initially confined to the anterior domain of the developing otic vesicle in *dlx3b/4b* mutants, but also expressed in a posterior patch at later stages (Fig. 5I,J and data not shown). Because *dlx3b/4b* mutants do not form functional early-born hair cells resulting in absence of otoliths, we also analyzed the expression of *otogelin* (*otog*). Otog can be found in the acellular membranes of the inner ear in zebrafish and amniotes (Lundberg et al., 2015). In addition, *otog* is required for proper seeding of otolith precursor particles in zebrafish (Stooke-Vaughan et al., 2015). Otolith precursor particles appeared in the otic vesicle at 18 hpf, and bind exclusively to the tips of the kinocilia of the first hair/early born cells or tether cells (Riley et al., 1997). We found that expression of *otog* could already be detected at OEPD and placodal stages in control, but not *dlx3b/4b* mutant, embryos (Fig. 5G,H and data not shown). At vesicle stages, expression of *otog* marked two domains at the poles of the otic vesicle in control siblings, whereas only the anterior domain was present in *dlx3b/4b* mutants (Fig. 5I,J). Taken together, these results show that only early-born hair cells (tether cells), which are required for otolith formation, are dependent on Dlx3b/4b activity. Later-forming hair cells develop through a Dlx3b/4b-independent mechanism. Furthermore, onset of *otog* expression in *dlx3b/4b* mutants is delayed but not sufficient to rescue otolith seeding.

### Increased OEPD-dependent neurogenesis in *dlx3b/4b* mutants

**Fig. 6.**
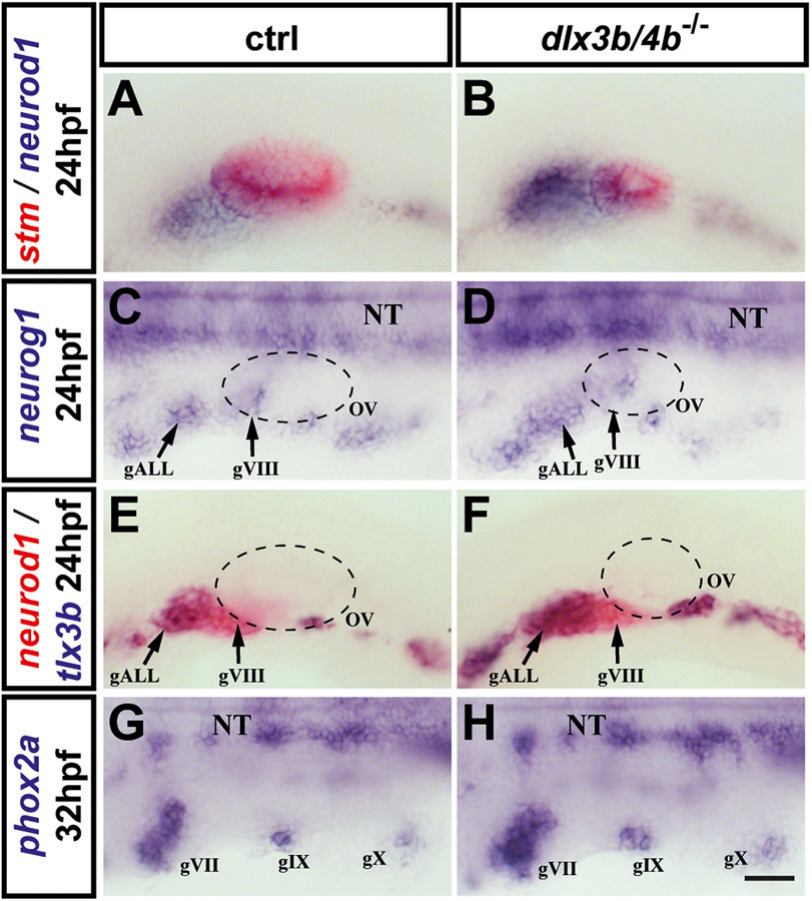
**OEPD-dependent neurogenesis in *dlx3b/4b*-deficient embryos.** (A,B) Expression of *neurod1* (blue) in control siblings and *dlx3b/4b* mutants at 24 hpf. Expression of *stm* (red) reveals the size reduction of the otic vesicle in *dlx3b/4b* mutants in comparison with wild-type embryos. (C,D) Expression of *neurog1* in control and *dlx3b/4b* mutant embryos at 24 hpf. (E,F) Double staining of *neurod1* (red) and *tlx3b* (blue) indicates increased production of anterior lateral line ganglion progenitors in *dlx3b/4b* mutants compared to control siblings at 24 hpf. (G,H) At 32 hpf, *phox2a* expression in *dlx3b/4b* mutants is indistinguishable from wild-type controls. (A,B,E,F) Lateral views with anterior to the left. (C,D,G,H) Dorsal views with anterior to the left. gALL, anterior lateral line ganglion progenitor; gVII, geniculate ganglion progenitor; gVIII, statoacoustic ganglion progenitor; gIX, petrosal ganglion progenitor; gX, nodose ganglion progenitor. Scale bar: 40 µm.

Previous studies have suggested that Dlx3b/4b play important roles in OEPD-derived neurogenesis (Millimaki et al., 2007; Dee et al., 2008; Padanad and Riley, 2011; Hans et al., 2013). To address this in the newly generated *dlx3b/4b* mutants, we examined *neurogenic differentiation 1* (*neurod1*), which is expressed in the anterior-ventral region of the otic vesicle, in delaminated neuroblasts as well as progenitors of the anterior lateral line ganglia, which are closely associated with the delaminated otic neuroblasts at 24 hpf (Andermann et al., 2002). In comparison with control embryos, *neurod1* expression increased in the absence of Dlx3b/4b, despite smaller otic vesicles indicated by starmaker (stm) expression (Fig. 6A,B). Increased *neurod1* expression in *dlx3b/4b* mutants is prefigured by increased levels of the proneural gene *neurogenin1* (*neurog1*), which delineates the neurogenic placodes including progenitors of the anterior lateral line ganglion (gALL) and cells within the otic vesicle that give rise to delaminated neuroblasts (gVIII) (Andermann et al., 2002) (Fig. 6C,D). Onset of *neurog1* expression, however, was unchanged and could be observed at 15 hpf similar to wild-type siblings (data not shown). To distinguish anterior lateral line progenitors and otic neuroblasts, we performed double labeling with *neurod1*, expressed in both lineages, and *T-cell leukemia, homeobox 3b* (*tlx3b*), which exclusively labels progenitors of anterior lateral line ganglion, but not otic neuroblasts, at 24 hpf (Langenau et al., 2002). In comparison with wild-type siblings, the majority of *neurod1*-labelled cells co-expressed *tlx3b* in *dlx3b/4b* mutants, indicating an increase in anterior lateral line progenitors in this genetic background (Fig. 6E,F). To examine any change in epibranchial ganglion progenitors, we analyzed the expression of *paired-like homeobox 2a* (*phox2a*) and *paired-like homeobox 2bb* (*phox2bb*), which label progenitors of the geniculate (gVII), petrosal (gIX) and nodose (gX) ganglion (Guo et al., 1999). In comparison with control siblings at 32 hpf, phox2a expression revealed that all epibranchial progenitor populations were initiated in a similar manner in both *dlx3b/4b*, which was confirmed by *phox2bb* expression at 50 hpf (Fig. 6G,H and data not shown). Taken together, these findings show that loss of Dlx3b/4b seems to result in defects in OEPD-derived neurogenesis with a potential increase in anterior lateral line ganglion progenitors. In contrast, otic and epibranchial placode neurogenesis seems to be unaffected and indistinguishable from that in the wild type in *dlx3b/4b* mutants.

## DISCUSSION

Their ease of use established morpholino oligonucleotides as a highly valuable knockdown tool in zebrafish, providing insights into various developmental mechanisms including inner ear development. Because reports indicated that several morpholino-induced phenotypes might be due to non-specific effects, morpholino oligonucleotides usage guidelines were established (Eisen and Smith, 2008). However, with the ability to apply reverse genetic approaches, a growing number of discrepancies emerged even in studies that largely adhered to the published usage guidelines. In a recent comprehensive study, >70% of morpholino-induced phenotypes failed to be observed in the corresponding mutants (Kok et al., 2015). Hence, it is now suggested that morpholino oligonucleotides should be no longer be considered a stand-alone tool for elucidating gene function in zebrafish. Consequently, appropriate mutant lines need to be generated and characterized to validate morpholino-induced phenotypes.

**Fig. 7.**
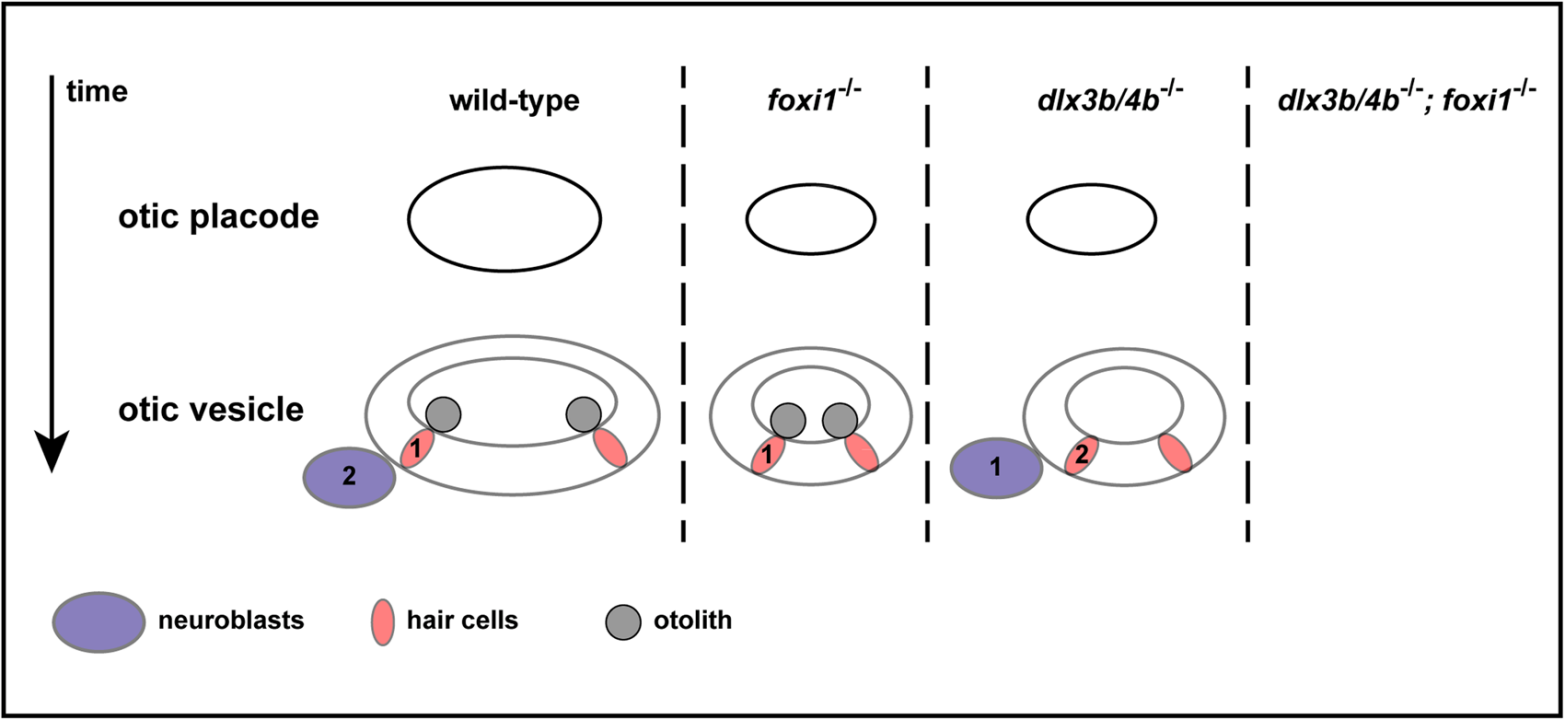
**Schematic of the events during early otic development in zebrafish.** In wild-type embryos, the otic placode develops into the otic vesicle with the formation of neuronal precursors/neuroblasts (purple) and sensory hair cells (light red) in a stereotypical temporal order. Sensory hair cells (1) are generated prior to the formation of neuroblasts (2). Subsequently, early-born hair cells (tether cells) seed and localize the formation of otoliths (grey). Loss of Foxi1 results in compromised otic induction and the development of smaller otic vesicles lacking the neuronal lineage. Loss of Dlx3b/4b also results in compromised otic induction and the development of smaller otic vesicles. However, otolith formation is completely abolished and the temporal order of neuronal and sensory hair cell formation is reversed. Combined loss of Dlx3b/4b and Foxi1 eliminates all indications of otic specification.

Here, we revisited the function of Dlx3b/4b in early inner ear development using CRISPR/Cas9-mediated mutagenesis. Removal of the entire *dlx3b* and *dlx4b* tail-to-tail bigene cluster resulted in the introduction of a defined deletion and the generation of an unambiguous null allele. We found that the deletion of the *dlx3b/4b* locus confirmed almost all previously reported results obtained with morpholino-mediated knockdown, showing that the published *dlx3b* and *dlx4b* morpholinos represent a reliable knockdown tool. Only reduced expression of *sox9a* in the OEPD after simultaneous morpholino-mediated knockdown of Dlx3b/4b (Liu et al., 2003) could not be confirmed. However, consistent with morpholino-mediated knockdown, mutant embryos showed a delayed onset of *pax2a*, resulting in compromised otic induction and subsequent otic vesicle hypoplasia (Fig. 7). Furthermore, combined loss of Dlx3b/4b and the forkhead transcription factor Foxi1 eliminated all indications of otic specification as previously described (Hans et al., 2004; Solomon et al., 2004). Our analysis of *dlx3b/4b* mutants also confirmed a role for Dlx3b/4b in sensorigenesis (Fig. 7). Otolith formation was defective in *dlx3b/4b* mutant embryos due to the absence of functional early-forming sensory hair cells or tether cells. In contrast, later-forming sensory hair cells were present and formed in a Dlx3b/4b-independent manner, indicating that distinct pathways control the development of early-born and later-forming sensory hair cells in zebrafish. We also found that loss of early-born sensory hair cells in *dlx3b/4b* mutant embryos reverses the common temporal sequence of neuronal and sensory hair cell specification in zebrafish (Fig. 7). Together with recent results from amniotes, our results demonstrate that vertebrate inner ear development is highly conserved but also suggest that zebrafish employ an additional Dlx3b/4b-dependent pathway, which has been either newly acquired in fish or lost in other vertebrate species. We propose that differences in otic induction, otolith versus otoconia formation, and the temporal order of neuronal and sensory hair cell specification result from the activity of the Dlx3b/4b-dependent pathway in fish. Expression of members of the distal-less gene family during early inner ear development has been reported in other species (Dirksen et al., 1993; Papalopulu and Kintner, 1993; Robinson and Mahon, 1994; Brown et al., 2005), but functional analysis suggests that otic placode induction in mammals and amphibians does not require distal-less gene function (Robledo et al., 2002; Saint-Germain et al., 2004). By contrast, expression analysis and functional characterization of Foxi3 in chick and mouse suggest that the amniote Foxi3 gene is the functional homolog of zebrafish foxi1 in otic and epibranchial placode formation (Edlund et al., 2015). Knockdown of Foxi3 causes a failure of otic placode induction in chick embryos (Khatri et al., 2014). Consistently, induction of the otic placode as well as formation of the otocyst completely fail to occur in Foxi3 mutant mice (Birol et al., 2016). Furthermore, Foxi3 is necessary for the development of the neurogenic epibranchial placodes similar to the role *foxi1* plays in zebrafish (Guo et al., 1999; Birol et al., 2016). Hence, in amniotes, loss of a single factor results in the complete loss of otic specification and is much more severe than changes seen in foxi1 mutant zebrafish. Here, only additional removal of the Dlx3b/4b-dependent pathway resulted in a complete block of otic development. Dlx3b/4b-dependent otic induction is required for the proper onset of *atoh1b*, which is necessary for the development of early-born hair cells (Millimaki et al., 2007; Hans et al., 2013). Early-born hair cells, or tether cells, are sensory hair cells that seed and localize the formation of otoliths, large solidified bio-crystals. Expression of a so-far unidentified hair cell-specific otolith precursor-binding factor is a key initiator of this process (Millimaki et al., 2007; Stooke-Vaughan et al., 2012). Consequently, in the absence of Dlx3b/4b function, early-born hair cells are lost or not properly specified, and otolith formation is completely absent. In contrast to zebrafish, other vertebrate species do not possess otoliths but rather form otoconia, minute biomineralized particles, which are anchored to the otoconial membrane atop the hair bundles (Lundberg et al., 2015). Because *dlx3b/4b* mutants failed to develop otoliths it would be interesting to determine if they instead form otoconia or otoconia-like structures. However, due to other defects, *dlx3b/4b* mutants are embryonic lethal around 5 days postfertilization, and a conditional *dlx3b/4b* allele will be required to address this question.

Loss of early-forming hair cells in *dlx3b/4b* mutant embryos reversed the temporal order of neuronal and hair cell specification. It has been reported that the order of cell specification in amniotes, *Neurog1* expression prior to *Atoh1* expression, is not constrained across phylogeny and that the temporal sequence of cell specification is reversed in zebrafish with *atoh1b* and *atoh1a* expression preceding *neurog1* expression (Millimaki et al., 2007; Fritzsch et al., 2010; Radosevic et al., 2014; Raft and Groves, 2015). Our results now indicate that two genetically distinct pathways result in the formation of early-born and later-forming sensory hair cells, respectively. The former are *atoh1b*-dependent whereas the latter are mostly dependent on *atoh1a*, as was previously shown using morpholino-mediated knockdown of Atoh1a and Atoh1b (Millimaki et al., 2007). Loss of Atoh1b activity following *atoh1b* morpholino-mediated knockdown, or as shown here in *dlx3b/4b* mutant embryos, delays onset of *atoh1a* expression beyond the onset of otic neurogenesis (Millimaki et al., 2007). Taken together, lack of the Dlx3b/4b-dependent pathway in zebrafish resembles events during amniotic inner ear development with respect to otic induction, absence of otoliths and temporal order of cell specification. Currently, it is unclear if the recruitment of the Dlx3b/4b pathway in early inner ear development is a feature in the ontogenesis of zebrafish, teleosts or all fish species. The spatio-temporal analysis of *Foxi1* and *Dlx3b* in medaka showed a large degree of conservation within the teleost lineage (Hochmann et al., 2007), but functional data are lacking. Future work will be needed to address this issue more rigorously.

In addition to its involvement in sensorigenesis, analysis of *dlx3b/4b* mutant embryos confirmed previous results that Dlx3b/4b participates in OEPD-dependent neurogenesis (Hans et al., 2013). These findings suggest that the early OEPD represents an equivalence group with progenitors of the inner ear and the anterior lateral line ganglion and that failure to enter the otic lineage results in the adoption of an anterior lateral line ganglion fate. In contrast, development of the third neuronal component of the OEPD, the epibranchial placodes, is unaffected by loss of Dlx3b/4b activity, supporting a model whereby the otic/anterior lateral line placodes form first followed by the subsequent induction of epibranchial placodes through an Fgf-relay (Padanad and Riley, 2011; Maulding et al., 2014).

## MATERIALS AND METHODS

### Ethics statement

All animal experiments were conducted according to the guidelines and under supervision of the Regierungspräsidium Dresden (permit AZ 24-9168.11-1/2013/29). All efforts were made to minimize animal suffering and the number of animals used. Heterozygous animals carrying the *dlx3b/4b* mutation do not show any obvious phenotype, and are indistinguishable from wild-type siblings during development and adulthood. In contrast, animals carrying the *dlx3b/4b* mutation in homozygosity are severely affected and highly lethargic at 120 hpf, although no gross anatomical malformations can be observed with the exception of the inner ear. Due to restrictions in our animal experimentation permits we cannot provide information about time of death and terminal phenotype because mutant larvae were always euthanized at 120 hpf.

### Cas9 and gRNA construction

Cas9 mRNA and gRNAs were synthesized as recently described (Jao et al., 2013). Briefly, Cas9 mRNA was synthesized by in vitro transcription using T3 mMESSAGE mMACHINE kit (Ambion, Austin, USA). gRNAs were generated and purified using the MEGAshortscript T7 and *mir*Vana miRNA isolation kits (Ambion), respectively. Sequences of the genomic target sites and oligonucleotides for making gRNAs are listed in Table 1.

**Table 1.**
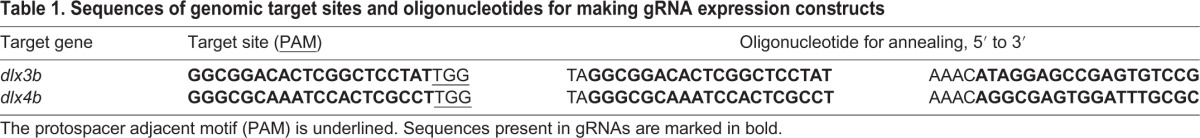
**Sequences of genomic target sites and oligonucleotides for making gRNA expression constructs**

### Zebrafish husbandry and germline transformation

Zebrafish were raised and maintained as previously described (Brand et al., 2002). Zebrafish embryos were obtained by natural spawnings of adult fish and staged according to hpf or standard criteria (Kimmel et al., 1995). The wild-type line used was AB. The transgenic line *Tg(pou4f3:GAP-GFP)*^s356^ has been described previously (Xiao et al., 2005). The mutation in *foxi1* (*foxi1^em1^*) including the genotyping protocol has been described by Solomon et al. (2003). For germ line transformation, Cas9 mRNA and gRNAs were co-injected into fertilized eggs, after which embryos were raised to adulthood, crossed to AB wild-type fish and the resulting F1 embryos were screened by PCR. To detect the deletion in the *dlx3b/4b* locus, the primers dlx3b/4b-1f 5′-CTGCTGATCGCTAAGGTTGTCTTCTGCC-3′ and dlx3b/4b-rev 5′-CTCCAGCATTTCACCTCTTCATTATCGCCATAAC-3′ were used, which result in an amplicon of 618 bp in the presence of the deletion allele. In total, 80 animals were screened and one founder carrying the *dlx3b/4b* deletion allele in the germline was identified. For subsequent genotyping, the primer dlx3b/4b-2f 5′-GGAGAGTCCTTAGAAGTCGGATGGCAACTC-3′ was added, amplifying a 473 bp fragment in the presence of wild-type DNA. Genotyping of embryos demonstrated the exact concordance between homozygous mutant genotype and observed phenotype. At least 12 mutant embryos from three separate clutches were analyzed for each experiment.

### *In situ* hybridization

cDNA probes that detect the following genes were used: *dlx3b* (Ekker et al., 1992), *pax2a* (Krauss et al., 1991), *pax8* (Pfeffer et al., 1998), *phox2a* (Guo et al., 1999), *myo7aa* (Ernest et al., 2000), *atoh1a* (Itoh and Chitnis, 2001), *neurog1* and *neurod1* (Andermann et al., 2002), *tlx3b* (Langenau et al., 2002), *sox9a* (Liu et al., 2003), *stm* (Söllner et al., 2003), *atoh1b* (Adolf et al., 2004) and *otog* (Stooke-Vaughan et al., 2015). Probe synthesis and *in situ* hybridization were performed essentially as previously described (Westerfield, 2000).

### Image acquisition, processing and statistical analysis

Images were acquired with a MVX10 microscope (Olympus, Hamburg, Germany), Axio Imager Z1 and Axiophot 2 microscope (Zeiss, Göttingen, Germany), and SP5 confocal microscope (Leica, Wetzlar, Germany) (in the light microscopy facility of the BIOTEC/CRTD at Technische Universität Dresden). All images shown are representatives of a fully penetrant phenotype. Images were processed with Adobe Photoshop or Fiji (https://imagej.nih.gov/ij/) and compiled with Adobe Illustrator.

The collected data were normally distributed and plotted as mean±s.e.m. Statistical significance was assessed using Mann–Whitney test in GraphPad Prism (*P<0.05; **P<0.01; ***P<0.001; ****P<0.0001). The sample size was ≥6 for each sensory patch.
